# Electrochemical Processes Coupled to a Biological Treatment for the Removal of Iodinated X-ray Contrast Media Compounds

**DOI:** 10.3389/fchem.2020.00646

**Published:** 2020-07-31

**Authors:** Wei Zhang, Isabelle Soutrel, Abdeltif Amrane, Florence Fourcade, Florence Geneste

**Affiliations:** ^1^Univ Rennes, CNRS, ISCR-UMR 6226, Rennes, France; ^2^Univ Rennes, Ecole Nationale Supérieure de Chimie de Rennes, CNRS, ISCR-UMR 6226, Rennes, France

**Keywords:** diatrizoate, electroreduction, deiodination, electrochemical oxidation, activated sludge, coupling process, biological treatment, biodegradability

## Abstract

Iodinated X-ray contrast media (ICM) compounds are a form of intravenous radiocontrast containing iodine, which are rapidly eliminated via urine or feces. The issue with the accumulation of ICM has received considerable critical attention since they are ubiquitously distributed in municipal wastewater effluents and in the aquatic environment and are not significantly eliminated by most biological sewage treatment processes. Among the methods that have been tested to eliminate ICM, electrochemical methods have significant advantages, since they can selectively cut the carbon-iodine bonds that are suspected to decrease their biodegradability. On the production sites, the recovery of iodine ions due to the carbon-iodine cleavage can be envisaged, which is particularly interesting to reduce the cost of the ICM production process. The coupling of an electrochemical process and a biological treatment can be carried out to mineralize the organic part of the formed by-products, allowing the recovery of the iodide ions. Therefore, the degradation of diatrizoate, a typical ionic ICM compound, by an electrochemical process was the purpose of this study. The electrochemical reduction of diatrizoate was performed using a flow cell with a graphite felt electrode at different potentials. The removal yield of diatrizoate reached ~100% in 2 h and the main product, 3,5-diacetamidobenzoic acid, was quantitatively formed, showing that diatrizoate was almost completely deiodinated. According to the BOD_5_/COD ratio, the biodegradability of diatrizoate after electrolysis was considerably improved. Cyclic voltammetry analysis of the electroreduced solution showed several oxidation peaks. The electrochemical oxidation of the by-products formed after the first treatment by electroreduction was then performed at three different potentials to study the influence of electrochemical oxidation on biodegradability. Results showed that the degradation yield of the deiodinated by-products increased with the potential and reached 100% at 1.3 V/SCE. Four different biological treatments were implemented during 21 days in stirred flasks with fresh activated sludge. The evolution of the mineralization during the biological treatment highlighted the biorecalcitrance of diatrizoate as previously estimated by the BOD_5_/COD ratio. Interestingly, the mineralization yield increased from 41 to 60% when electrochemical oxidation at 1.3 V/SCE was implemented after electroreduction.

**Graphical Abstract:**
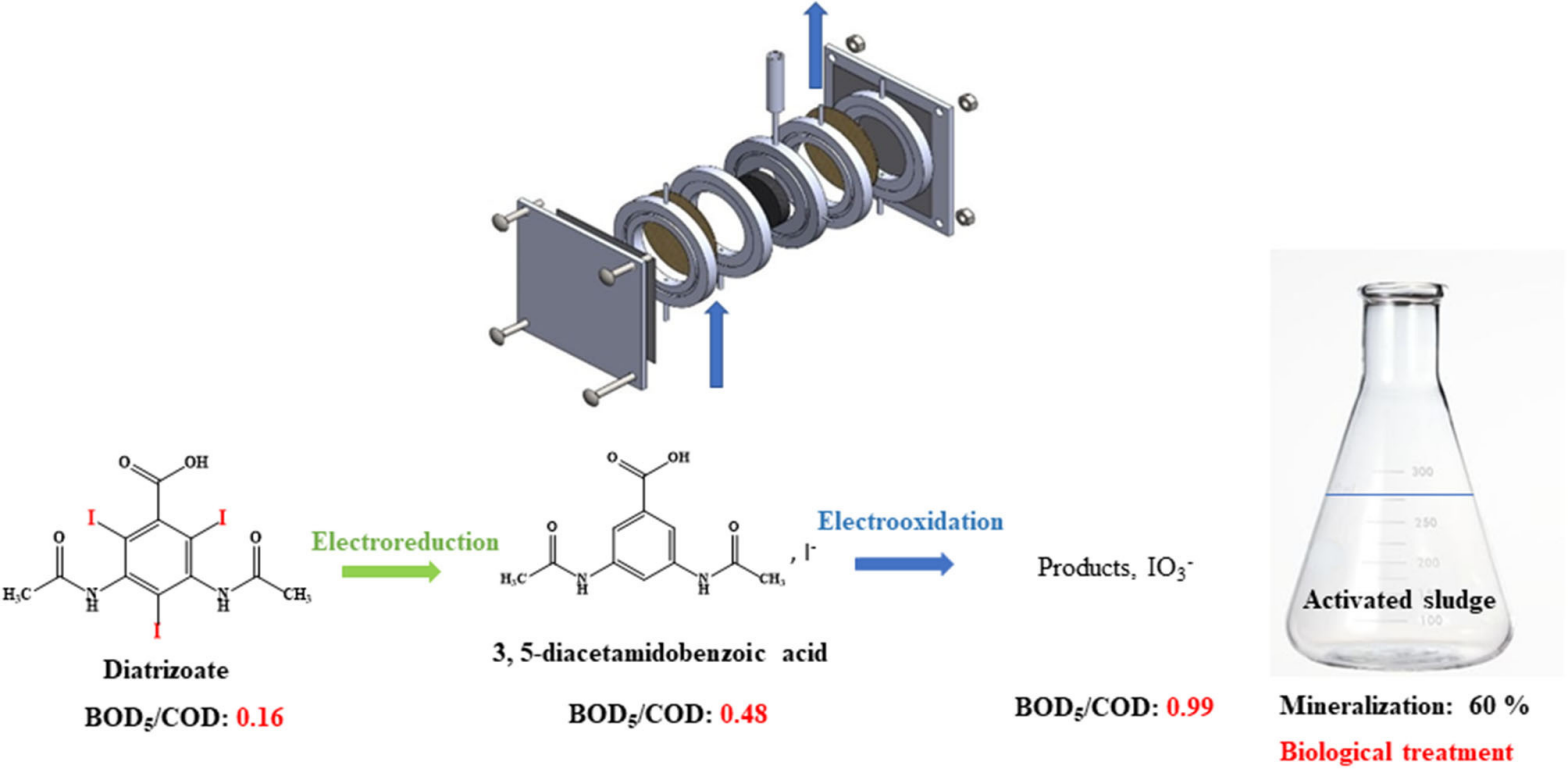
Electrochemical reduction and then oxidation of diatrizoate allow total deiodination and improvement of the biodegradability of the solution leading to a mineralization yield of 60%.

## Introduction

Since the application of X-ray on medical diagnostic imaging examination, iodinated X-ray contrast media (ICM) have been extensively used as contrast agents due to their ability to enhance the visibility of image. ICM belong to the derivatives of 2,4,6-triiodobenzoic acid, which usually contain polar carboxyl, hydroxyl or amide moieties on their side chains. Three iodine atoms were introduced on the benzene ring for X-ray adsorption; meanwhile other polar groups were added to assure their high water solubility and resistance to human body metabolism (Yu and Watson, 1999; Estep et al., 2000). ICM are routinely administered with huge dose (up to 200 g per patient for single checking). Hence, each year the global consumption of ICM reaches up to the impressive amount of 3,500 tones. Furthermore, ICM are commonly eliminated from patient's body by urine and feces after 24 h (Steger-Hartmann et al., 1999; Pérez and Barceló, 2007). Therefore, ICM have been frequently detected in hospital effluents, domestic sewage systems as well as drinking water at relatively high concentrations. For example in the downstream of Danube river in Germany, more than 500 ng L^−1^ of diatrizoate acid and iopamidol were found (Seitz et al., 2006), whereas in a Spain Sewage Treatment Plant, the concentration of iopromide was in the range 6.60–9.30 μg L^−1^ (Carballa et al., 2004) and organic iodine concentration in Texas in the United States, varied between 5 and 40 μg L^−1^ (Drewes et al., 2001). Although ICM ecotoxicology still needs to be further confirmed, many researchers have claimed that ICM are transformed into toxic by-products during drinking water disinfection process (Jeong et al., 2017).

Regarding this emerging environmental issue, various approaches have been applied for the removal or degradation of ICM in the past two decades. These approaches include biological treatment, advanced oxidation processes (AOPs) and reductive deiodination. Owing to their cost-effectiveness in terms of investment and operating cost, biological treatments have been performed directly on ICM, focusing on the identification of transformation products (Haiß and Kümmerer, 2006; Kormos et al., 2010; Redeker et al., 2014). However, the biodegradability of ICM is very low owing to their particular molecular structure, which strictly restricts their metabolization under aerobic or anaerobic conditions. Although AOPs such as ozonation, UV based radicals, namely hydroxyl sulfate or chloride radicals, and plasma have shown high ability to degrade ICM, the formation of toxic intermediates has been observed, leading to the need for the development of alternative processes (Huber et al., 2005; Duan et al., 2017; Zhou et al., 2017; Banaschik et al., 2018; Hu et al., 2019).

The removal of ICM through reductive deiodinations has been consequently envisaged as more selective methods (Zwiener et al., 2009; Mu et al., 2011; Stieber et al., 2011; Radjenovic et al., 2013; Korshin and Yan, 2018; Yan et al., 2018). Among them, electroreduction reaction can be considered as a green technique to treat pollutants since the addition of a chemical reducing agent is not required. It is also known as an economic and effective method due to its high selectivity for dehalogenation of organic halide pollutants (Fontmorin et al., 2014a; He et al., 2016, 2017, 2018; Verlato et al., 2016; Lou et al., 2019, 2020), allowing the recovery of iodide ions. Thus, the reductive deiodination of ICM has been applied for the removal of iopamidol with the aim to form controlled intermediates (Yan et al., 2018). Nevertheless, the fate of released iodine and the post-treatment of deiodination products have been only little explored (Zwiener et al., 2009; Radjenovic et al., 2013; Yan et al., 2018).

**Scheme 1:**
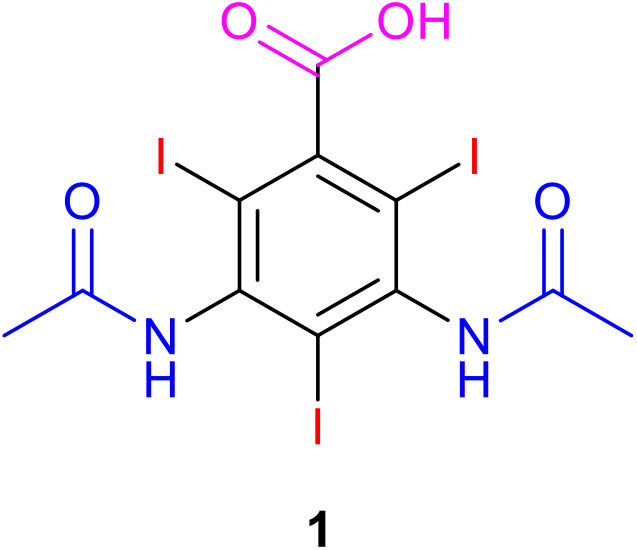
Structure of diatrizoate **1**.

To associate the benefit of a selective electrochemical processes and low expensive biological treatments (Assassi et al., 2011; Fontmorin et al., 2014b; Belkheiri et al., 2015; Saidi et al., 2017; Zaghdoudi et al., 2017; Geneste, 2018), we investigated the coupling of both methods for the removal of the highly recalcitrant contrast agent, diatrizoate (DTR) (Scheme 1). Graphite felt was used as a cost-effective porous electrode with high specific surface area in a flow-through system to improve the mass transfer, decrease the electrolysis time, and facilitate the industrial application of the electrochemical process. First, total deiodination of diatrizoate was performed in a flow electrochemical cell containing a graphite felt electrode. To complete this first electrochemical step, the deiodination products, which were electroactive in oxidation, were then electrochemically oxidized at different potentials. The biodegradability of the electrolyzed solutions after the different treatments were compared. Finally, a biological treatment by activated sludge was performed to complete the degradation process.

## Materials and Methods

### Chemicals and Materials

All chemicals used in this work were of analytical grade. Anhydrous sodium sulfate (Na_2_SO_4_) 99%, sodium diatrizoate acid **1** and 3,5-diacetamidobenzoic acid were purchased from Sigma-Aldrich (France). Graphite felt (GF, Recycled vein graphite RVG 4000) was obtained from Mersen (France). Dimensionally stable anodes (DSA), AC-2004, were supplied by ECS International Electro Chemical Services, France.

### Electrochemical System

**Figure 1:**
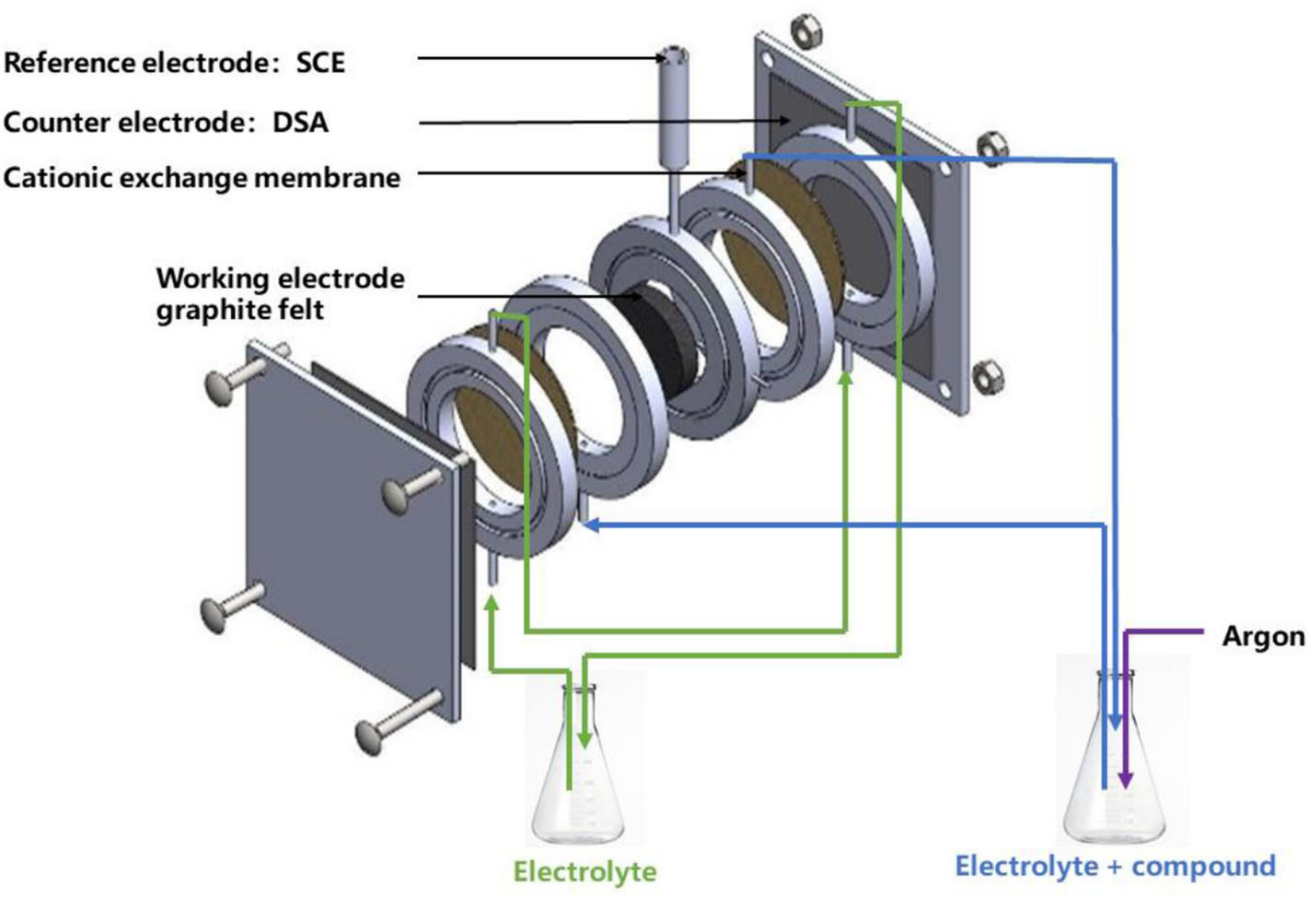
Flow electrochemical cell.

The electrochemical flow cell (Figure 1) was constructed with two equal ring-shaped Perspex frames with internal diameter 4.8 cm and thickness 1.6 cm. A cation exchange membrane (Nafion^TM^ 417, France) was inserted in the middle of two glue-sealed single frames. Two unit frames, two counter electrodes (DSA) and a working electrode (graphite felt diameter 4.8 cm and thickness 1.0 cm) were bolted together between two plastic plates. The total empty volumes for cathodic compartment and anodic compartment were both 29 mL. Before use graphite felt was dipped in an ultrasonic bath of ethanol for 30 min, then rinsed 10 times with deionized water to eliminate ethanol. The working electrode, counter electrode, and reference electrode were connected through a potentiostat (VersaSTAT 3 from Elancourt, France). The electrolytic solution flowed through the graphite felt with recycling.

### Cyclic Voltammetry Analysis (CV)

CV analyses were performed with an EDAQ potentiostat unit equipped with the ECchem solfware package. The electrochemical experiments were implemented in a typical three electrodes system with a saturated calomel electrode (SCE) as the reference electrode, a platinum plate as a counter electrode and a glassy carbon (*d* = 0.25 cm) as the working electrode. The working electrode was carefully polished with sand paper (European # P4000, Struers, Ballerup, Denmark) and rinsed with distilled water before each run. All experiments were conducted in argon saturated solutions at room temperature.

### HPLC Analysis

The residual diatrizoate and the concentration of 3,5-diacetamidobenzoic acid were measured by high performance liquid chromatography (HPLC). Samples were analyzed after filtering through a 0.2 μm membrane filter. The HPLC system consisted of a Waters 996 High Performance Liquid Chromatography equipped with a Waters 996 PDA (Photodiode Array Detector) and a Waters 600 LCD Pump. The separation was conducted on a column Waters C-18 (5 μm; 4.6 × 250 mm). 10 μl of sample were injected with an auto-sampler. The carrier liquid consisted of the solution A composed of 95% of deionized water and 5% acetonitrile as well as 0.1% formic acid, and the solution B composed of acetonitrile containing 0.1% formic acid (A 100%, B 0% for 2 min, then a gradient to A 0% B 100% in 6 min and for 2 min and finally a gradient to A 100% B 0% in 1 min at a flow rate of 0.4 mL min^−1^). Diatrizoate and 3,5-diacetamidobenzoic acid were detected at a wavelength of 238 nm at a column temperature of 35°C at 1.09 and 4.20 min, respectively.

### Ion Chromatography Analysis

I^−^ and IO_3−_ ions were measured by an ion chromatography (IC) system [DIONEX DX120 equipped with a conductivity detector and a DIONEX AS19 (4 × 250 mm) ion-exclusion column]. The sample was eluted with potassium hydroxide at a flow rate of 1 mL min^−1^. The detection was analyzed by conductivity with a Self-Regenerating Suppressor (SRS).

### Biodegradability Evaluation

The biodegradability was evaluated by the ratio of Biological Oxygen Demand in 5 days (BOD_5_) to Chemical Oxygen Demand (COD), which was performed in an incubator box set (Oxitop IS6; WTW, Ales, France) at 20°C (Fontmorin et al., 2014b). The biodegradable ability of the DTR, of its products of electrochemical reduction (ER), as well as of the electrochemical oxidation (EO) process was determined. Activated sludge (AC), taken from the Beaurade wastewater treatment plant (Rennes, France), was considered. Before treatment, activated sludge had to be washed 8–10 times to remove the dissolved organic matter. For BOD_5_ test, samples were added in 500 mL brown bottles containing the culture medium with orbital shaking (300 rpm). The composition of the BOD_5_ culture medium was: MgSO_4_·7H_2_O, CaCl_2_, FeCl_3_·6H_2_O, NH_4_Cl, KH_2_PO_4_, K_2_HPO_4_ at concentrations of 22.5, 27.5, 0.15, 2.0, 6.8, 2.8 g L^−1^, respectively. The concentration of activated sludge was 0.05 g L^−1^. All samples were adjusted to pH 7 before BOD_5_ measurement and at least duplicated. Then bottles were closed with sensor caps, which automatically recorded each hour the BOD_5_ value. A carbon dioxide scrubber containing solid sodium hydroxide was fixed above the solution.

### Biological Treatment

Biological treatment was conducted in aerobic mode, in a 500 mL Erlenmeyer flask with activated sludge at an initial concentration of 0.5 g L^−1^. The composition of the culture medium was: MgSO_4_·7H_2_O, CaCl_2_, FeCl_3_·6H_2_O, NH_4_Cl, Na_2_HPO_4_·2H_2_O, KH_2_PO_4_, K_2_HPO_4_ at concentrations of 22.6, 27.6, 0.26, 75, 154.4, 85, 208 g L^−1^, respectively. Experiments were performed in an incubator with orbital shaking (250 rpm) at 25°C for 21 days. Samples were taken at an interval of 2 or 3 days for total organic carbon (TOC) determination, to calculate the mineralization yield. The TOC values were read from a Total Organic Analyzer Shimadzu TOC-VCPH/CPN with the method of standard NPOC (Non-Purgeable Organic Carbon).

## Results and Discussion

### Electrochemical Reduction of Diatrizoate

#### Cyclic Voltammetry Analysis

**Figure 2:**
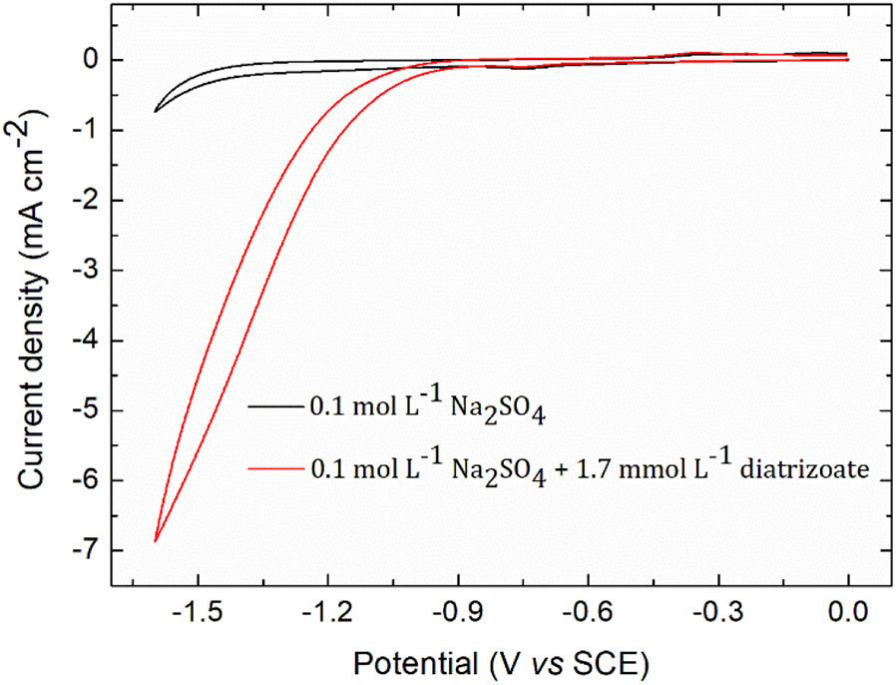
Cyclic voltammogram of 1.7 mmol L^−1^ diatrizoate in 0.1 mol L^−1^ Na_2_SO_4_ on glassy carbon electrode. Scan rate 0.1 V s^−1^.

The electroactivity of diatrizoate was determined by cyclic voltammetry analysis (CV) on glassy carbon electrode in 0.1 mol L^−1^ Na_2_SO_4_ (Figure 2). The onset potential corresponding to the reduction of diatrizoate was −1.1 V/SCE (−0.43 V vs. RHE). The absolute current density increased for potentials lower than −1.1 V/SCE, although hydrogen evolution was observed in the blank at around −1.4 V/SCE (−0.75 V vs. RHE). From these analyses, electrochemical reduction of diatrizoate was tested at different potentials ranging from −1.1 V to −1.5 V/SCE to find the optimal conditions.

#### Effect of Applied Potentials

**Figure 3:**
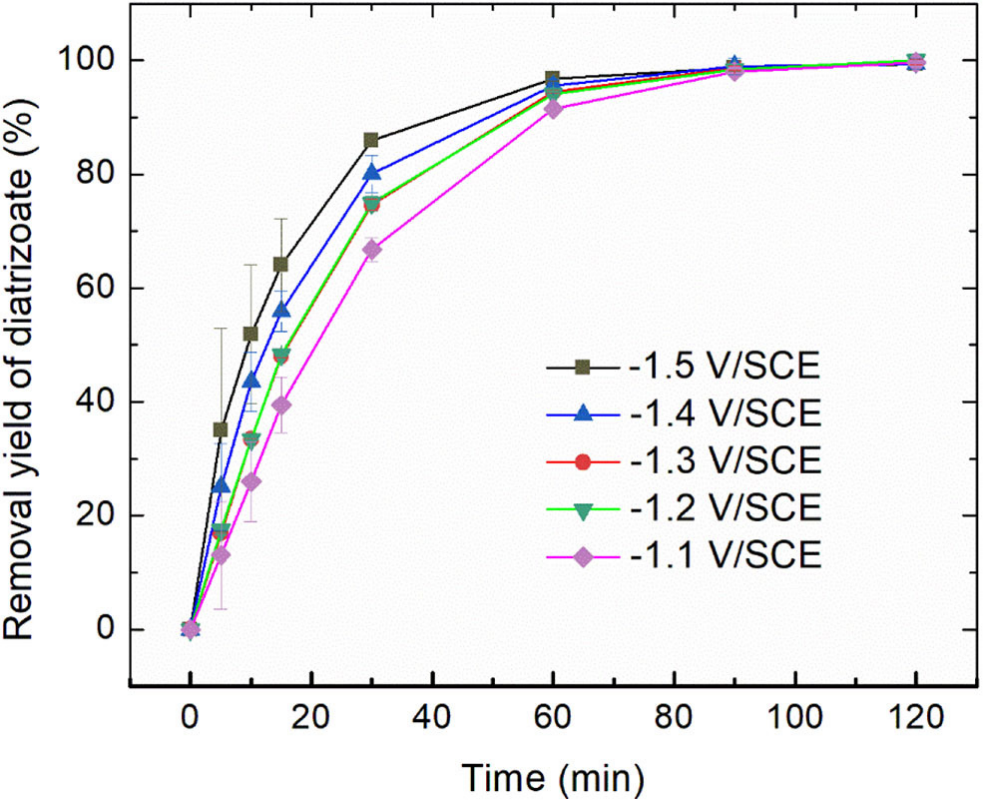
Removal yield of diatrizoate (100 mg L^−1^ in 0.1 mol L^−1^ Na_2_SO_4_, 100 mL) vs. time during electroreduction in the electrochemical flow cell (3 mL min^−1^). Error bars are based on duplicate experiments.

Electroreduction of diatrizoate on graphite felt electrode was performed in a flow electrochemical cell at 5 different potentials ranging from −1.1 to −1.5 V/SCE. The removal yield of diatrizoate was estimated by HPLC analysis during electrolysis (Figure 3).

The kinetic of the electrolysis increased when the potential was more negative and reached a maximum for −1.4 and −1.5 V/SCE.

**Figure 4:**
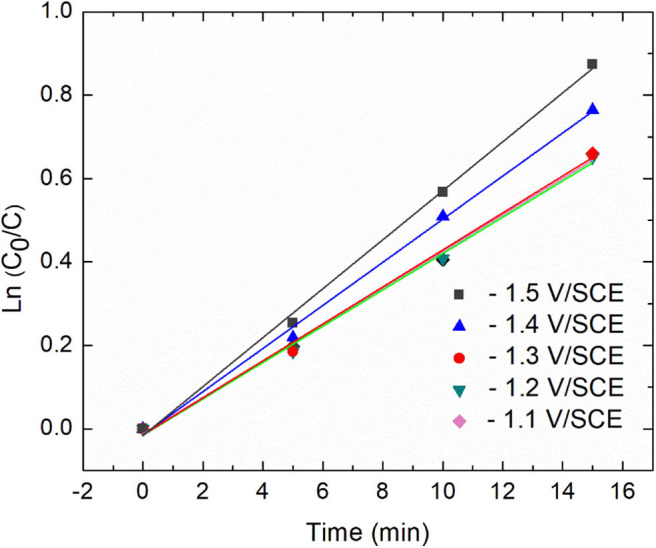
Relationship between diatrizoate removal yield and time during 15 min electroreduction for five different applied potentials.

Additional insights about the kinetic were derived from the plots of ln[C_0_/C] against time (Figure 4).

Diatrizoate deiodination followed a first-order kinetic model for all tested potentials according to Equation (1), with regression coefficients *R*^2^ higher than 0.99 (Table 1):

(1) $$\begin{array}{l} -\frac{d\left[DTR\right]}{d\;t}=k_{obs}{\left[DTR\right]}_{t} \end{array}$$

Where k_obs_ (min^−1^) is the observed pseudo-first-order rate constant. The value of k_obs_ increased from 0.044 to 0.059 min^−1^ (around 30%) as the applied potential decreased from −1.1 to −1.5 V/SCE. However, the removal yield of diatrizoate was close to 100% after 2 h electrolysis for all studied potentials.

**Table 1:** Value of k_obs_ and *R*^2^ for each studied potential.

	**−1.1 V**	**−1.2 V**	**−1.3 V**	**−1.4 V**	**−1.5 V**
k_obs_ (min^−1^)	0.044	0.044	0.044	0.052	0.059
*R*^2^	0.996	0.997	0.996	0.996	0.998

**Figure 5:**
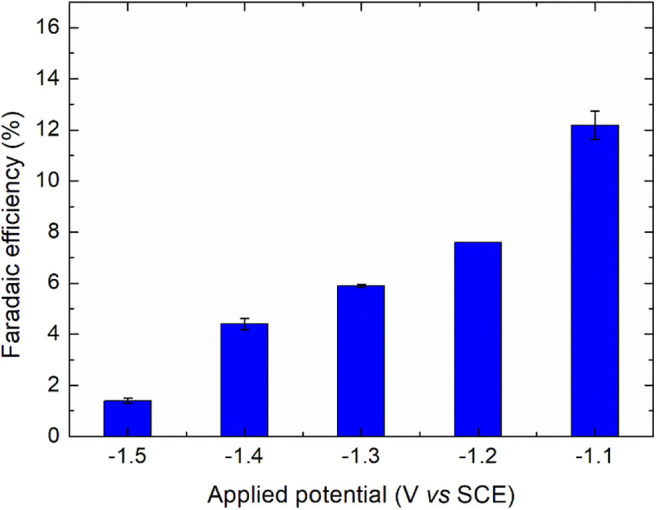
Faradaic efficiencies for the electroreduction of diatrizoate at applied potentials ranging from −1.1 to −1.5 V/SCE. Error bars are based on duplicate experiments.

To complete the comparison, the faradaic efficiency of diatrizoate reduction ε was calculated considering three reductive deiodination reactions as follows:

(2) $$\begin{array}{l} \varepsilon =\frac{3\times 2\times V\times {\left[DTR\right]}_{i}\times F}{Q} \end{array}$$

where 2 is the number of electrons involved in the deiodination reaction, *F* is the Faraday's constant (96 485 C mol^−1^), *[DTR]*_*i*_ is the initial concentration of diatrizoate (mol L^−1^), *V* is the volume of solution (L) and *Q* is the electric charge consumed during electrolysis (C). As observed in Figure 5, the faradaic efficiency increased when the potential was less negative, attributed to a competition of the reaction with hydrogen evolution. It is also worth noting that the contribution of the residual current in the faradaic efficiency was high due to the low concentration of diatrizoate used in the flow electrochemical cell. An optimization of the cell would allow the improvement of the faradaic efficiency.

Hydrogen evolution was confirmed by an increase of the solution pH beyond 11 for all studied potentials. Further experiments were performed with a potential of −1.3 V/SCE, which represents a good compromise between the kinetic of the reaction and the faradaic efficiency.

#### Mass Balance of Diatrizoate Electroreduction

**Figure 6:**
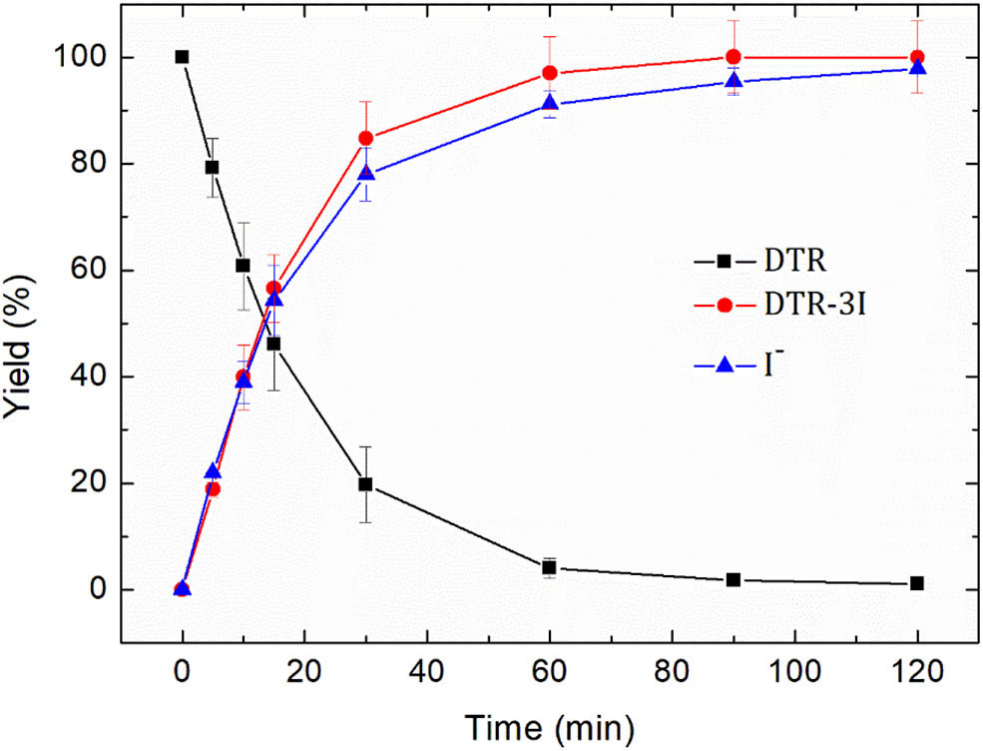
Removal yield of diatrizoate (DTR), yield of 3,5-diacetamidobenzoic acid (DTR-3I) and deiodination yield (I^−^) during electroreduction of diatrizoate (100 mg L^−1^) at −1.3 V/SCE.

The electroreduction of diatrizoate (100 mg L^−1^) was conducted on graphite felt in 0.1 mol L^−1^ Na_2_SO_4_ solution at −1.3 V/SCE. The amount of released iodide ions was measured by ion chromatography and the concentration of the deiodinated derivative 3,5-diacetamidobenzoic acid (DTR-3I) by HPLC with a commercially available standard. As observed in Figure 6, the decrease of the diatrizoate concentration with time was accompanied by an increase of both the deiodination yield and the yield of 3,5-diacetamidobenzoic acid.

After 2 h electroreduction, diatrizoate was almost completely removed, whereas 3,5-diacetamidobenzoic acid was formed quantitatively and the deiodination yield reached 97.8 ± 0.01%. These results underline the high selectivity of the electroreduction process to cut the carbon-iodine bonds.

The effectiveness of the electroreduction process can be also underlined. Indeed, whereas a partial electroreduction of diatrizoate on graphite felt has been previously noticed with remaining iodinated intermediates even after 2 h electrolysis (Radjenovic et al., 2013), a total deiodination was obtained in this work. This enhanced activity is probably due to the used flow electrochemical cell, which favors mass transport through the felt. Therefore, the easily adaptability of the process to large-scale treatments and its high selectivity and effectiveness makes it promising as a pretreatment of DTR with possible iodide ions recovery (Radjenovic et al., 2013) before a coupling with a biological treatment.

### Electrochemical Oxidation of Deiodination Products

#### Cyclic Voltammetry Analysis

**Figure 7:**
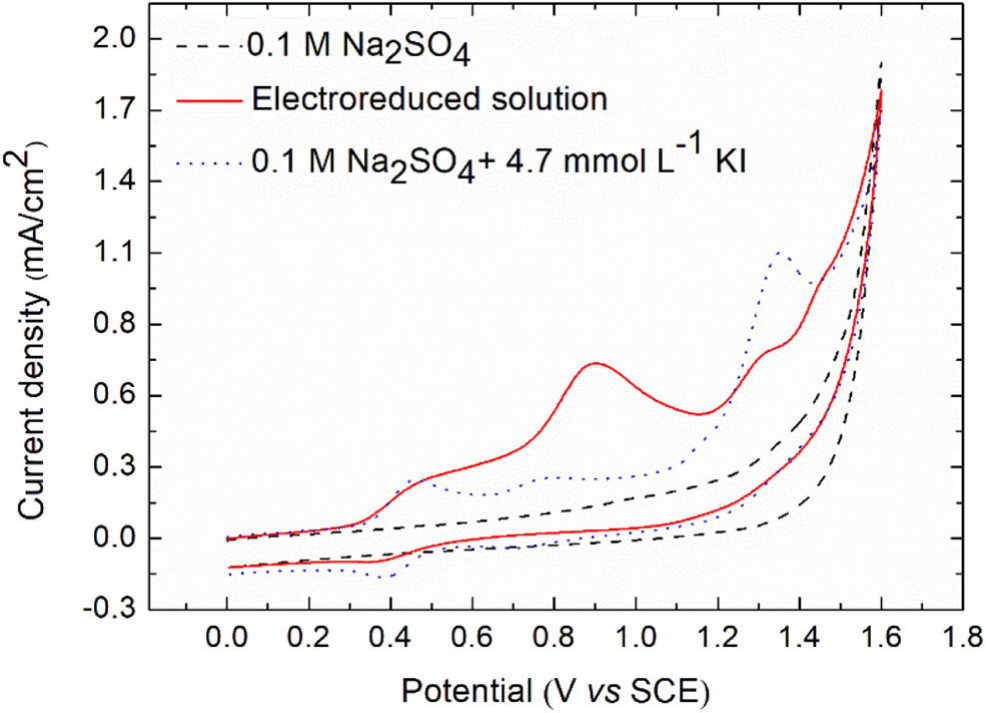
Cyclic voltammetry of electrolytic medium after electroreduction and of potassium iodide for comparison on glassy carbon electrode. Scan rate: 0.1 V s^−1^.

After electroreduction of diatrizoate in 0.1 mol L^−1^ Na_2_SO_4_ at −1.3 V/SCE for 2 h, the electrolytic medium was analyzed by cyclic voltammetry (Figure 7).

As expected, no peak was observed in negative potentials. However, four oxidation peaks were noticed at 0.5, 0.9, 1.3, and 1.45 V/SCE. To check if these peaks were due to the oxidation of iodide ions released after electroreduction of DTZ, a solution of 4.7 × 10^−3^ mol L^−1^ KI in 0.1 mol L^−1^ Na_2_SO_4_, corresponding to the concentration of iodide ions released after electroreduction was analyzed. A first reversible system was observed at 0.42 V/SCE, corresponding to Equation (3).

(3) $$\begin{array}{l} {\text{I}}_{2}\;+\;2\text{e}-⇌2{\text{I}}^{-} \end{array}$$

Two other irreversible oxidation peaks appeared at 0.79 and 1.35 V/SCE, which were supposed to correspond to the oxidation peaks of the electrolyzed solution observed at 0.9 and 1.45 V/SCE, due to the difference of pH of both solutions (around 1–2 units). The first one probably corresponded to the oxidation of iodine into iodate IO_3−_ and the last one to the formation of higher-oxidation-state iodinated compounds (Pourbaix, 1966). Therefore, the peak at 1.3 V/SCE could be due to the oxidation of 3,5-diacetamidobenzoic acid. To confirm this hypothesis, the electrochemical oxidation of the electroreduced solution was performed at 0.5, 0.9, and 1.3 V/SCE.

**Figure 8:**
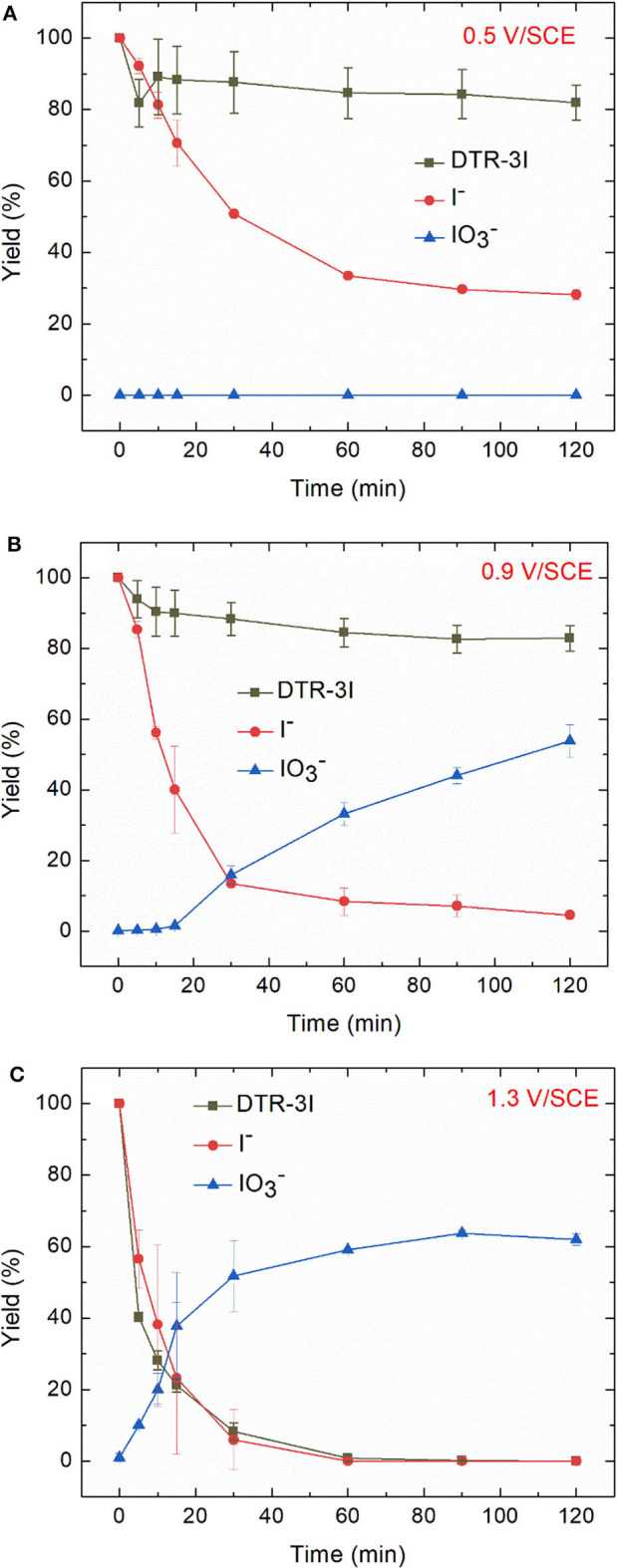
Electrochemical oxidation of electroreduced diazoate solution (100 mg L^−1^ in 0.1 mol L^−1^ Na_2_SO_4_ at −1.3 V/SCE for 2 h) at **(A)** 0.5 **(B)** 0.9 V, and **(C)** 1.3 V/SCE. Error bars are based on duplicate experiments.

Figure 8 displays the evolution of the removal yield of DTR-3I and I^−^ during electrochemical oxidations performed at 0.5, 0.9, and 1.3 V/SCE. At 0.5 V/SCE, the concentration of iodide ions decreased, showing their oxidation into iodine, whereas no iodate IO_3−_ were formed. When the applied potential was 0.9 V/SCE, ion chromatography analysis revealed an increase of the degradation yield of iodide ions and the presence of a new peak, identified as iodate IO_3−_. However, the electrochemical oxidation of DTR-3I was still low. At 1.3 V/SCE, DTR-3I was quantitatively oxidized, confirming that the peak at 1.31 V/SCE observed in cyclic voltammetry analysis was due to the oxidation of DTR-3I. Iodide ions were also totally oxidized, whereas the yield of IO_3−_ was 62 ± 2%.

#### Biodegradability Estimation

**Figure 9:**
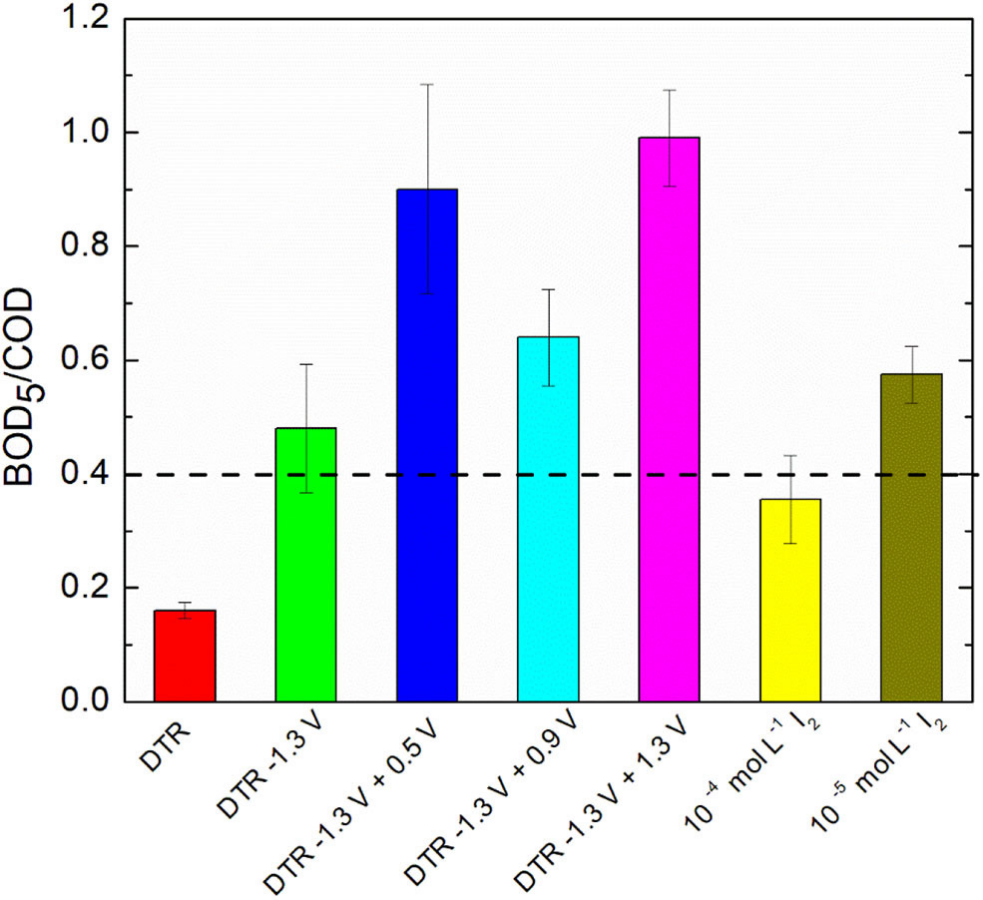
BOD_5_/COD ratio of DTR before and after electrochemical treatments and I_2_ solutions in 0.1 mol L^−1^ Na_2_SO_4_. Error bars are based on duplicate experiments.

After the electrochemical treatment of diatrizoate, the biodegradability was evaluated considering the BOD_5_/COD ratio. Indeed, a solution is considered as biodegradable when this ratio is higher than 0.4 (Lou et al., 2020). After electroreduction, the BOD_5_/COD ratio was enhanced from 0.16 ± 0.02 to 0.48 ± 0.11 (Figure 9), which was just above the threshold level of biodegradability.

Since electrochemical oxidation of iodide ions led to the formation of iodine, which is well-known for its antiseptic properties, the influence of I_2_ concentration on biodegradability was first evaluated. The theoretical maximum concentration of I_2_, c_I__2__T, M___, can be calculated according to the following equation, considering only IO_3−_ and I_2_ as oxidation products of iodide ions:

(4) $$\begin{array}{l} {\text{c}}_{{\text{I}}_{{\text{2}}_{\text{T},\text{M}}}}={\text{c}}_{{\text{I}-}_{\text{R}}}-{\text{c}}_{{\text{I}-}_{\text{D}}}-{\text{c}}_{{\text{IO}}_{{\text{3}}_{\text{D}}^{-}}} \end{array}$$

Where, c_I−_R__ is the concentration of released I^−^ after electroreduction, c_I−_D__ the detected concentration of I^−^ after electrochemical oxidation and ${\text{c}}_{{\text{IO}}_{{\text{3}}_{\text{D}}^{-}}}$ the concentration of detected IO_3−_ after electrochemical oxidation.

Thus, the theoretical maximum concentrations of I_2_ after electrochemical oxidation at 0.5, 0.9 and 1.3 V/SCE were 0.8 × 10^−5^, 0.14 × 10^−5^, and 6.2 × 10^−5^ mol L^−1^, respectively. Therefore, the impact of I_2_ on biodegradability was examined for two I_2_ concentrations, 10^−4^ and 10^−5^ mol L^−1^ in 0.1 mol L^−1^ Na_2_SO_4_. The BOD_5_/COD ratio was close to 0.4 for both concentrations (Figure 9), showing that the presence of I_2_ in the electrolyzed solution should not affect the biodegradability measurements. Furthermore, after electrochemical oxidations, the solutions were degassed overnight under argon to decrease their concentration in I_2_.

Therefore, the biodegradability after electrochemical oxidations at the different potentials was estimated with the BOD_5_/COD ratio (Figure 9). A significant increase of the ratio was observed for all potentials and reached a maximum of 0.90 ± 0.08 for electrolysis performed at 1.3 V/SCE, showing that adding an electrochemical oxidation step after the electroreduction of diatrizoate is in favor of a coupling with a biological treatment. However, the BOD_5_/COD ratio was significantly lower for oxidations performed at 0.9 V/SCE. It could be explained by the presence of iodate ions in the solution cumulated with DTR-3I. Although careful washing of the activated sludge was performed, the presence of remaining carbon substrate could impact the intrinsic values of the BOD_5_/COD ratios. To confirm these first results, a biological treatment was therefore considered.

#### Biological Treatment

**Figure 10:**
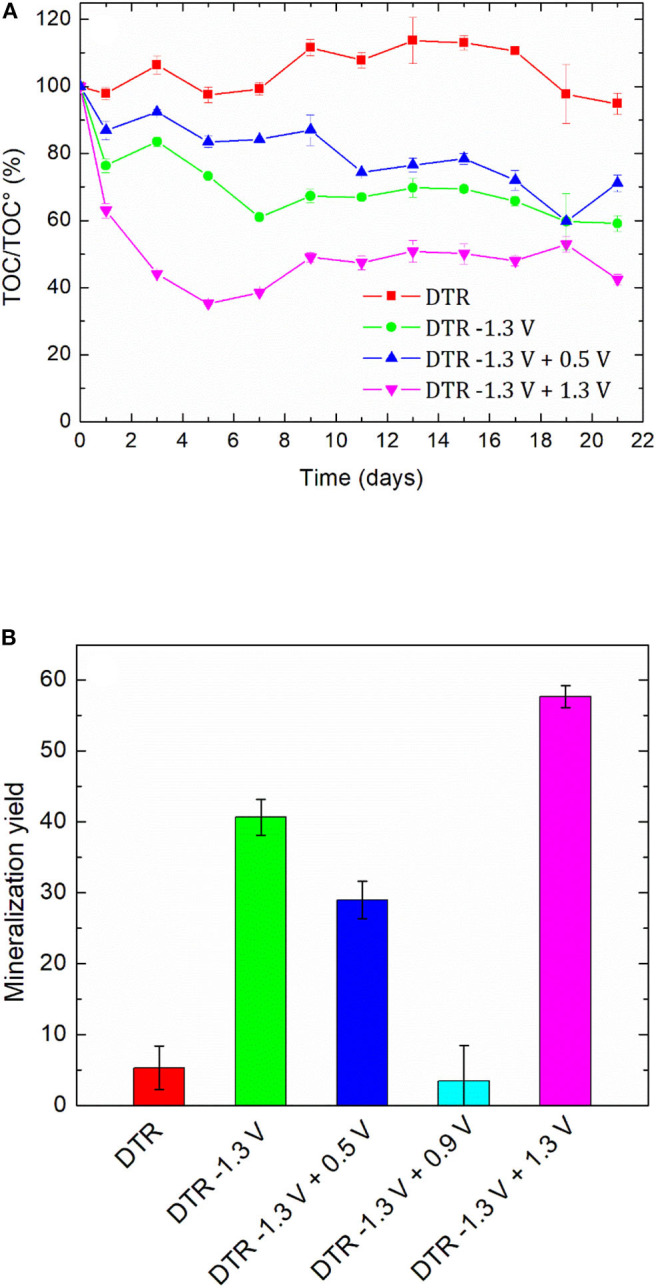
**(A)** Time-courses of TOC values and **(B)** final mineralization yield during activated sludge culture on DTR solution (100 mg L^−1^, 0.1 mol L^−1^ Na_2_SO_4_) before and after electrochemical treatments. Error bars are based on triplicate experiments.

The high values of BOD_5_/COD ratio led us to perform a biological treatment for the non-treated solution, namely 100 mg L^−1^ DTR to check the biorecalcitrance of this compound, the solutions after an electroreduction at −1.3 V/SCE and coupled with an electrochemical oxidation step at 0.5, 0.9, and 1.3 V/SCE. All the oxidized solutions were degassed under argon overnight before the biological treatment. The degradation of diatrizoate was monitored by TOC measurement for 21 days of activated sludge culture (Figure 10). As displayed in Figure 10A, the mineralization of DTR remained very low with only 5% decrease, confirming its biorecalcitrance. This result is consistent with other studies, showing that DTR is difficult to remove by biological treatment owing to its poor biodegradability (Haiß and Kümmerer, 2006). After DTR deiodination by electroreduction at −1.3 V/SCE, a significant improvement of the mineralization yield was observed, which slowly increased until 41% after a 21 days culture period. When a subsequent electrochemical oxidation treatment was performed at 0.5 V/SCE, the biodegradability of the solution was similar to those obtained after electroreduction. The 10% oxidation of DTR-3I and the low concentration of iodine in solution did not influence significantly the biodegradability of the solution. After the electrochemical oxidation at 0.9 V/SCE, the biodegradability of the solution was even lower. This behavior was consistent with the trend given by the BOD_5_/COD ratio (Figure 9), although the intrinsic value >0.6, showing a good biodegradability of the solution was not confirmed by the biological treatment (Figure 10B). Concerning the presence of iodate ions during biological treatment, literature studies highlighted their influence on bacteria growth, as it has been shown for *Escherichia coli* (Wang et al., 2006). Indeed, it has been observed that for IO_3−_ concentrations lower than 6.67 mM, iodate ions had a significant impact on bacteria metabolism and for higher concentrations, a cellular lysis occurred as well as growth inhibition. However, some specific microorganisms such as *Shewanella oneidensis* (Toporek et al., 2019), *Shewanella putrifaciens*, and *Desulfovibrio desulfuricans* (Councell et al., 1997) are able to use iodate ion as final electron acceptor in anaerobic conditions. In the anoxic conditions of the biological treatment performed in this study, it is difficult to specify the impact of iodate ions on the microbial culture. Since electrolyses performed at 0.5 and 0.9 V/SCE exhibited similar concentrations of DTR-3I (Figures 8A,B), and the concentration of iodate was the same for electrolyses performed at 0.9 and 1.3 V/SCE (Figures 8B,C), we suspected the simultaneous presence of DTR-3I and iodate ions to be responsible for the lower biodegradability. Best results were obtained with a coupling between electroreduction at −1.3 V/SCE and electrochemical oxidation at 1.3 V/SCE, leading to a mineralization yield of 60% after only 5 days of culture. The oxidation of DTR-3I promoted biodegradability, compared with electrolysis performed at 0.9 V/SCE. However, refractory organic carbon (around 42%) still remained even after 21 days of activated sludge culture. The amido groups on the side chain of the benzene ring may be responsible of the persistence of the by-products, as previously observed with other pollutants (Lou et al., 2020).

## Conclusions

This work focused on the mineralization of diatrizoate, a biorecalcitrant X-ray contrast agent. For this purpose, an electrochemical pretreatment was performed to improve its biodegradability before a biological process to complete its mineralization. Electroreduction of diatrizoate led selectively to its total deiodination product, 3,5-diacetamidobenzoic acid. A total electrochemical oxidation of 3,5-diacetamidobenzoic acid was also achieved at 1.3 V/SCE. A first estimation of the biodegradability of the solutions considering the BOD_5_/COD ratio showed an improvement of biodegradability after all studied electrochemical treatments. A biological treatment with activated sludge was therefore considered on a period of 21 days. As expected, a low mineralization yield (5%) was obtained for the non-treated diatrizoate solution. This value increased to 41% after electroreduction and to 60% after the entire electrochemical treatment combining electroreduction with a subsequent electrochemical oxidation step performed at 1.3 V/SCE. These results show that the coupling of selective electrochemical treatments with a biological process is a promising cost-effective approach for diatrizoate removal.

Since the amido groups attached to the benzene ring are suspected to be responsible of the presence of refractory organic carbon after the biological treatment, a catalytic electroreduction of diatrizoate is now under consideration to reduce both the carbon-iodide bond and the amido groups.

## Data Availability Statement

The original contributions presented in the study are included in the article/supplementary materials, further inquiries can be directed to the corresponding author/s.

## Author Contributions

AA, FF, and FG developed the concept and designed the experiments. WZ carried out the experiments, interpreted the results, and prepared the manuscript. IS supervised the chromatographic analyses. AA, FF, and FG reviewed the manuscript. All authors approved it for publication.

## Conflict of Interest

The authors declare that the research was conducted in the absence of any commercial or financial relationships that could be construed as a potential conflict of interest.
